# First-Line Aumolertinib in *EGFR*-Mutant Advanced Non-Small Cell Lung Cancer: A Multicenter Real-World Retrospective Study with a Four-Year Follow-Up

**DOI:** 10.32604/or.2025.064119

**Published:** 2025-08-28

**Authors:** Xi Qin, Yulan Liu, Lin Zhu, Yunyan Mo, Jing Zhang, Zhuchun Jiang, Dongning Huang, Xinrong Hu, Jingzhang Li, Quanfang Chen, Feng Xue

**Affiliations:** 1 Department of Oncology, Affiliated Hospital of Guilin Medical University, Guilin, 541001, China; 2 Department of Radiotherapy, Affiliated Hospital of Guilin Medical University, Guilin, 541001, China; 3 Department of Oncology, Guilin People’s Hospital, Guilin, 541300, China; 4 Department of Oncology and Integrated Traditional Chinese and Western Medicine, The People’s Hospital of Hezhou, Hezhou, 542899, China; 5 Department of Oncology, Liuzhou Workers’ Hospital, Liuzhou, 545001, China; 6 Department of Oncology, Nanxishan Hospital of Guangxi Zhuang Autonomous Region, Guilin, 541002, China; 7 Department of Oncology, Liuzhou People’s Hospital, Liuzhou, 545001, China; 8 Department of Respiratory and Critical Care Medicine, The First Affiliated Hospital of Guangxi Medical University, Nanning, 530021, China

**Keywords:** Aumolertinib, non-small cell lung cancer (NSCLC), safety, epidermal growth factor receptor (EGFR), real world

## Abstract

**Background:**

The use of third-generation different tyrosine kinase inhibitors (TKIs) is considered the most effective option for treating advanced non-small cell lung cancer (aNSCLC) with epidermal growth factor receptor (EGFR) mutations. However, there is limited information on the efficacy and safety of aumolertinib in patients remains these cases.

**Methods:**

The clinical records of patients receiving aumolertinib as first-line therapy across four hospitals in the Guangxi Zhuang Autonomous Region from April 2020 to December 2021 were retrospectively analyzed, using progression-free survival (PFS) as the primary endpoint and overall survival (OS) representing the secondary endpoint. Adverse events (AEs) were assessed using the Common Terminology Criteria for Adverse Events (CTCAE v5.0).

**Results:**

Approximately 47 patients with *EGFR*-Mutant aNSCLC were recruited, including 1 squamous cell carcinoma (SCC) patient, 1 *EGFR* G719C mutated patient, 1 *EGFR* S768 patient mutated, and 1 *EGFR* KDD mutated patient. The average follow-up duration was 48.1 months concluding in August 2024. The median PFS (mPFS) was 22.2 months (95% CI 17.6 to 26.7), while the median OS (mOS) was 39.7 months (95% CI 32.6 to 46.9). Patients with deletion of exon19 in *EGFR* (19del) showeda mPFS of 28.4 months, markedlylonger than those with the L858R point mutation (L858R), who had a mPFS of 15.2 months (*p* = 0.036). Overall, 22 patients (46.8%) had central nervous system (CNS) metastases at the basal level. The mPFS for this cohort was 19.7 months. Rashes (17.0%), skin decrustation (4.2%), pruritus (4.2%), dental ulcers (4.2%), increased creatine kinase (2.1%), and musculoskeletal pains (2.1%) were the most prevalent AEs in this study. Grade 3 and higher AEs were observed at a rate of 4.2%.

**Conclusion:**

This study concluded that aumolertinib has considerable safety and efficacy for *EGFR*-mutant NSCLC in a first-line defense.

## 1 Introduction

Lung cancer is a highly aggressive and prevalent disease globally, with approximately 2.2 million new cases and 1.8 million deaths reported in 2020 [1]. In 2022, Chinese government statistics indicated approximately 1,060,600 new cases of LC [2]. Its elevated incidence rate signifies a major threat to public health and a substantial societal burden. Traditional treatment for LC is of paramount significance. However, NSCLC is the predominant pathophysiological subtype of LC, and its survival rates have markedly improved due to targeted therapy and immunochemotherapy [3,4]. Mutations in the EGFR are the predominant category of mutations in NSCLC, and various tyrosine kinase inhibitors (TKIs) have been prescribed for individuals with *EGFR*-sensitive mutations [5]. Besides, 1st or 2nd-generation TKIs show more effectiveness relative to platinum-based chemotherapy. However, acquired resistance (AR) to these generations of TKIs is unavoidable. *EGFR* T790M is the predominant resistance mechanism in secondary *EGFR* mutations [6]. The third-generation TKI, osimertinib, has been found both effective and safe in treating NSCLC patients with *T790M* and *EGFR*-sensitive mutations [7–9]. Osimertinib has received approval from theUS FDA for treating cases of *EGFR* T790M-positive aNSCLC showing disease progression with first- and second-generation TKIs, as well as treatment-naïve cases with *EGFR* sensitizing mutations. This approval marks the onset of a new era in third-generation targeted therapies.

Aumolertinib is classified as a 3rd generation TKI and functions as an oral, irreversible therapeutic agent that explicitly targets both *EGFR*-sensitizing and *T790M* mutations [10]. In the study of AENEAS involving advanced *EGFR* mutated-positive NSCLC Chinese patients, their mPFS was reported as 19.3 months for aumolertinib relative to 9.9 months for gefitinib. Moreover, AEs of grade > 3 severity (regardless of cause) occurred in 36.4% of patients treated with aumolertinib and 35.8% of those receiving gefitinib [11]. However, OS data were not disclosed in this study. Among the existing 3rd generation TKIs, only osimertinib has revealed OS data, with a mOS of 38.6 months [9]. Commutations frequently lead to unfavorable prognoses. Tumor protein *53* (*TP53*) is the most frequent variant among numerous co-mutations [12,13]. Further, phase III clinical research trials cannot accurately depict real-world scenarios due to the stringency of their inclusion and exclusion criteria.

This study reports a multicenter clinical trial evaluating the effectiveness of aumolertinib as a first-line therapy in treatment-naïve cases diagnosed with *EGFR*-mutant aNSCLC.

## 2 Materials and Methods

### 2.1 Study Population

**Figure 1:**
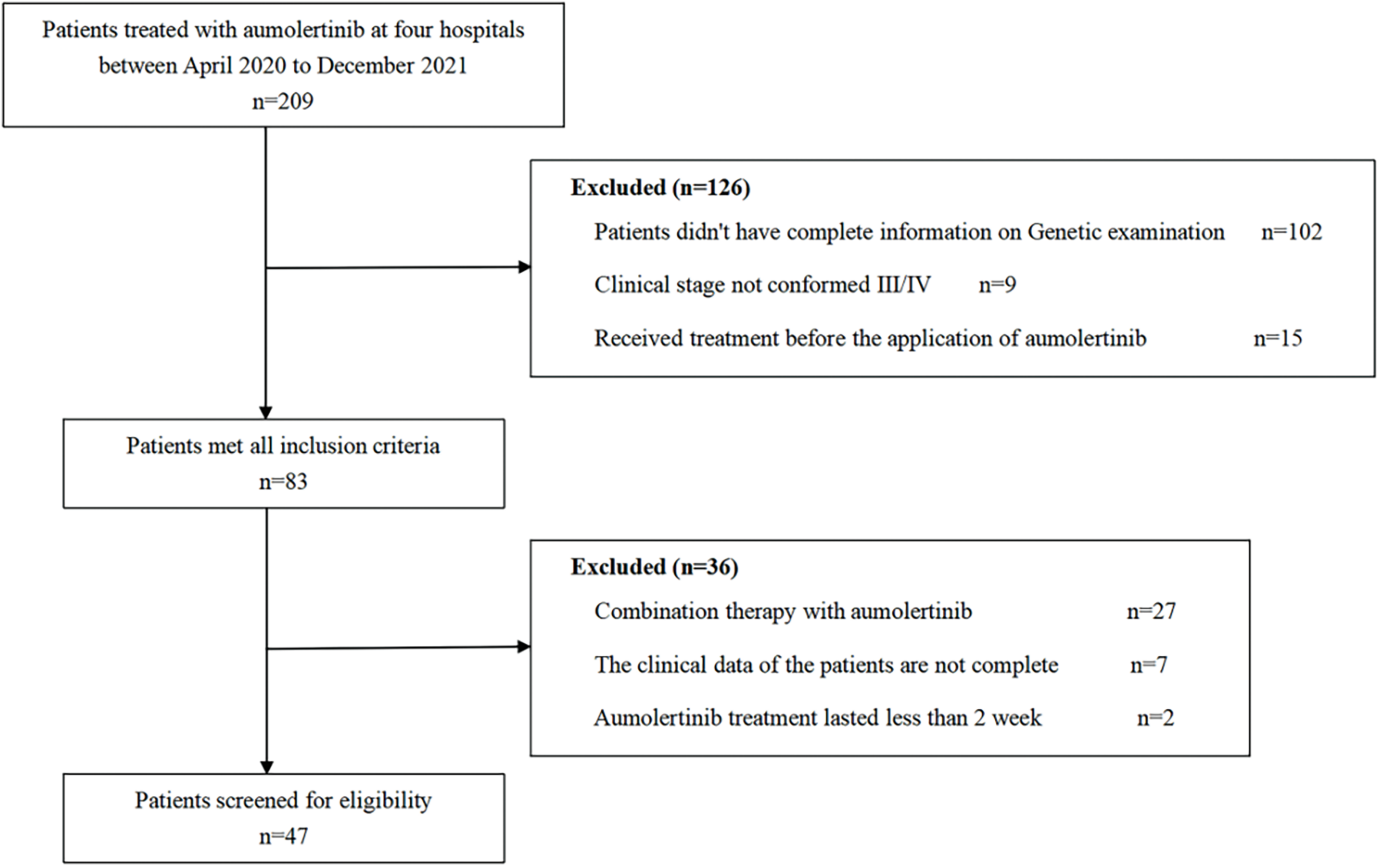
Flowchart depicting patient participation in the study

Patients with aNSCLC who were administered aumolertinib at 4 hospitals in the Guangxi Zhuang Autonomous Region of China between April 2020 and December 2021 were recruited for this retrospective analysis. Inclusion criteria: (1) Pathologically confirmed NSCLC; (2) Confirmation of *EGFR* mutations via polymerase chain reaction (PCR) or next-generation sequencing (NGS); (3) Clinical stage IIIB or IV; (4) Age ≥ 18 years; (5) No prior treatment before initiation of aumolertinib; (6) Presence of measurable lesions as described in the Response Evaluation Criteria in Solid Tumors (RECIST v1.1) [14]. The exclusion criteria were: (1) combined aumolertinib therapeutics; (2) incomplete medical history; (3) aumolertinib treatment lasting < 2 weeks. All screening procedures are illustrated in Fig. 1. From the medical records of each patient, data on several clinicopathological factors, such as sex, age, histopathological subtype, clinical grading, diagnosis date, distant metastasic site, and information about key genes (type of *EGFR* mutation, associated mutations in NGS), were obtained. This retrospective study was approved by the Medical Ethics Committee of the Affiliated Hospital of Guilin Medical University (Guilin, China; approval no. 2022IITLL-05-G1), which fully complies with the Declaration of Helsinki. Each patient or their closest relatives submitted an informed consent. The study report follows the STROBE guidelines to enhance the standardization of the paper.

### 2.2 Drug Administration

All eligible patients received 110 mg oral aumolertinib once per day as 1st-line treatment until progression of the disease as per the RECIST v1.1 [14], death, or unacceptable toxicity. All AEs were examined via the Common Terminology Criteria for Adverse Events (CTCAE 5.0).

### 2.3 Assessment of Efficacy and Toxicity

The primary and secondary outcomes were PFS and OS, respectively. The examiner assessed all endpoints or outcomes in line with RECIST. Furthermore, PFS represented the interval between initiation of aumolertinib treatment to disease progression or death, while OS was measured from treatment start to death. The subsequent phase included obtaining survival information through telephone interviews or visits to an ambulatory clinic and hospitalization data up to 30 August 2024.

### 2.4 Statistical Analysis

Data was statistically examined via SPSS^®^ software v27.0 (IBM Corp., Armonk, NY, USA). All survival curves were generated via the Kaplan-Meier method. The log-rank test was used to compare differences in PFS between the groups. The PFS differences between groups were compared using the log-rank test. Cox proportional hazards regression models were employed to assess the influence of various variables on PFS. A double tail with a *p* < 0.05 defined the level of significance.

## 3 Results

### 3.1 Basic Information about Patients

Forty-seven cases treated at 4 hospitals in China between April 2020 and December 2021 were recruited. The respondent population comprised 27.7% males and 72.3% females, aged 58 years on average (range 41 to 87 years). Pathologically, there was one case of lung SCC and 46 cases of adenocarcinoma. All diagnoses with stage IV.

**Figure 2:**
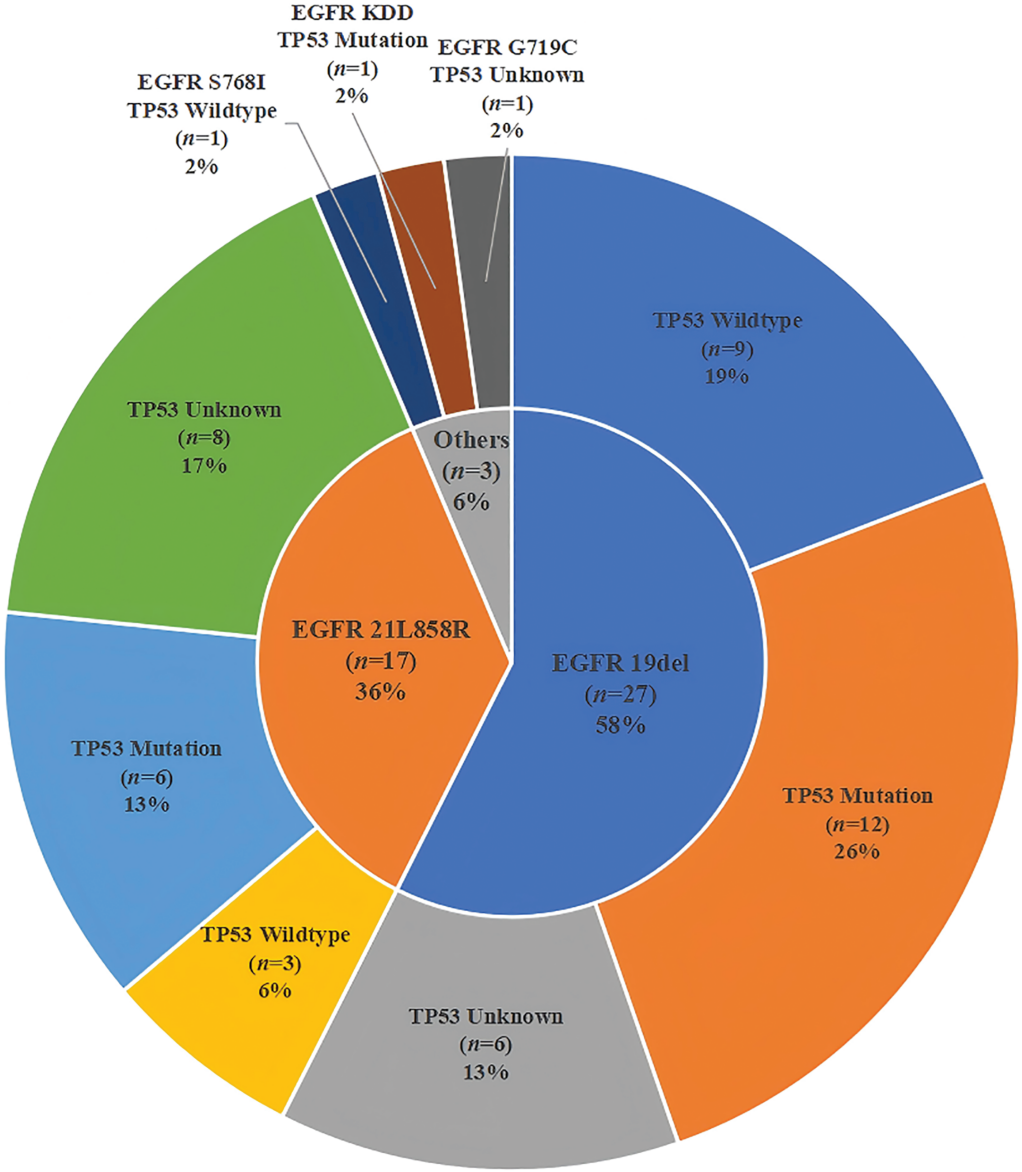
Pie chart illustrates the distribution of *EGFR* mutated and *TP53* mutated status in patients

**Figure 3:**
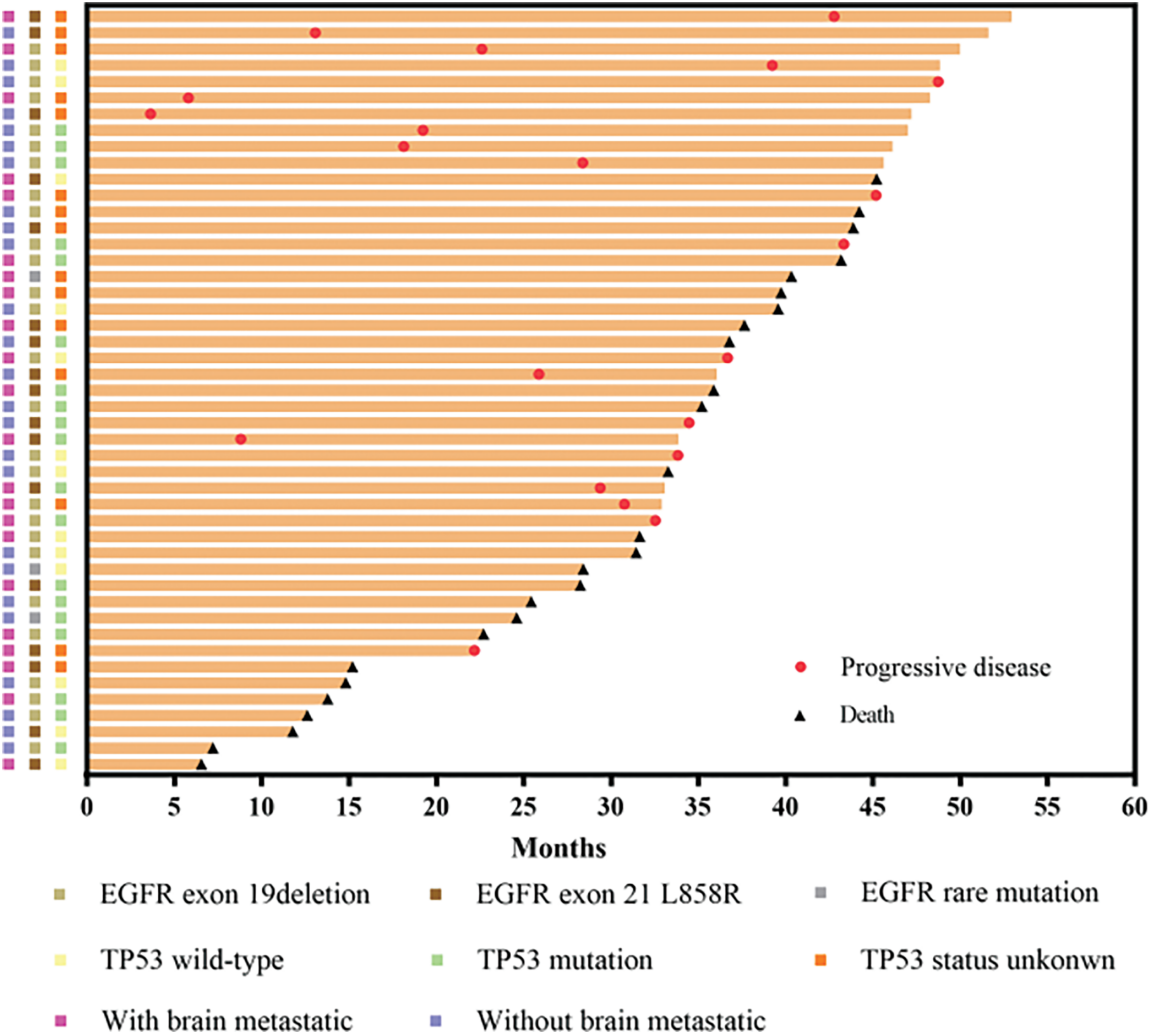
Swimmer plot showing length of cancer control in cases with different *EGFR* mutations and *TP53* status, as well as with and without CNS metastasis

Both NGS and PCR analyses of these patients revealed that 57.4% (27/47) of them carried the 19del, 36.2% (17/47) had the L858R, and 6.4% (3/47) contained other types of *EGFR* variations. Meanwhile, the proportion of *TP53*-positive patients (32/47, 68.1%) was higher than the *TP53*-negative patients (15/47, 31.9%). The proportions of *TP53* mutation, wild-type (WT) or unknown mutations in patients with 19del, *L858R*, or the other *EGFR* mutations, including *G719C, KDD*, and *S768I*, were different (Fig. 2). Fig. 3 illustrates the clinical treatment strategies for patients categorized by their respective gene mutations. Among all patients, 46.8% (22/47) had brain metastasis, 40.4% (19/47) had bone metastasis, and 46.8% (22/47) had lung or pleural metastasis (Table 1).

**Table 1:** Basic demographic and disease traits of patients

		Number of patients (%) *n* = 47
Sex		
	Male	13 (27.7)
	Female	34 (72.3)
Age, years	Median	58 (41–87)
	≤58 years	25 (53.2)
	>58 years	22 (46.8)
Smoking history		
	Yes	8 (17.0)
	No	39 (83.0)
Site of metastasis		
	CNS	22 (46.8)
	Bone	19 (40.4)
	Lung or pleura	22 (46.8)
Gene mutation status		
	EGFR exon 19del	27 (57.4)
	EGFR exon 21 L858R	17 (36.2)
	Others	3 (6.4)
TP53 status		
	Wild-type	13 (27.7)
	Mutation	19 (40.4)
	Unknown	15 (31.9)
Best overall response		
	Complete response	0 (0)
	Partial response	36 (76.6)
	Stable disease	9 (19.1)
	Progressive disease	2 (4.3)
ECOG performance status		
	0	24 (51.1)
	1	17 (36.2)
	2	6 (12.7)
Comorbidities		
	Cardiovascular Disease	9 (19.1)
	Diabetes Mellitus	6 (12.8)
	COPD	6 (12.8)

Note: CNS, central nervous system; EGFR, epidermal growth factor receptor; ECOG, Eastern Cooperative Oncology Group Performance Status; COPD, chronic obstructive pulmonary disease.

### 3.2 Efficacy of Treatment

**Figure 4:**
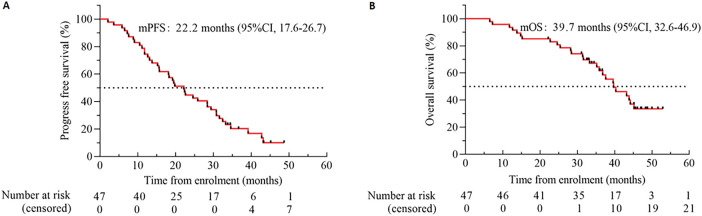
Kaplan-Meier estimates of PFS **(A)** and OS **(B)**. Black tick marks illustrate censored data. Abbreviation: CI denotes confidence interval

**Figure 5:**
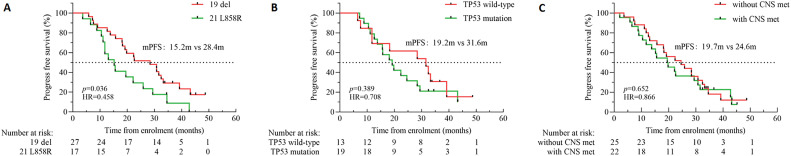
Kaplan-Meier survival curves representing the following parameters: PFS; **(A)** PFS in patients with the *EGFR* exon 19del or the *L858R* mutation; **(B)** PFS in patients with the *TP53* WT or *TP53* mutation; **(C)** PFS in patients with or without CNS metastases

The investigator assessed the mPFS at 22.2 months (95% CI 17.6 to 26.7) (Fig. 4A) and the mOS at 39.7 months (95% CI 32.6 to 46.9) (Fig. 4B). The standard follow-up duration was 48.1 months by the end of August 2024, and 44.7% (21/47) of the patients were recovered. The mPFS of cases with the 19del mutation was 28.4 months (95% CI 14.1 to 42.6), markedly longer than the 15.2 months (95% CI 10.0 to 20.4) seen in those with L858R (*p* < 0.05) (Fig. 5A). The mPFS of *TP53* WT patients was 31.6 months (95% CI 15.0 to 48.3), and that of *TP53* mutated cases was 19.2 months (95% CI 13.4 to 25.1). Both groups failed to show a substantial variation in mPFS (*p* = 0.389) (Fig. 5B). The mPFS was 19.7 months (95% CI 11.5 to 27.5) for all cases with CNS metastases. In contrast to patients who did not have CNS metastases, the mPFS was 24.6 months (95% CI 13.8 to 35.4, HR: 0.866; *p* = 0.652) (Fig. 5C).

**Figure 6:**
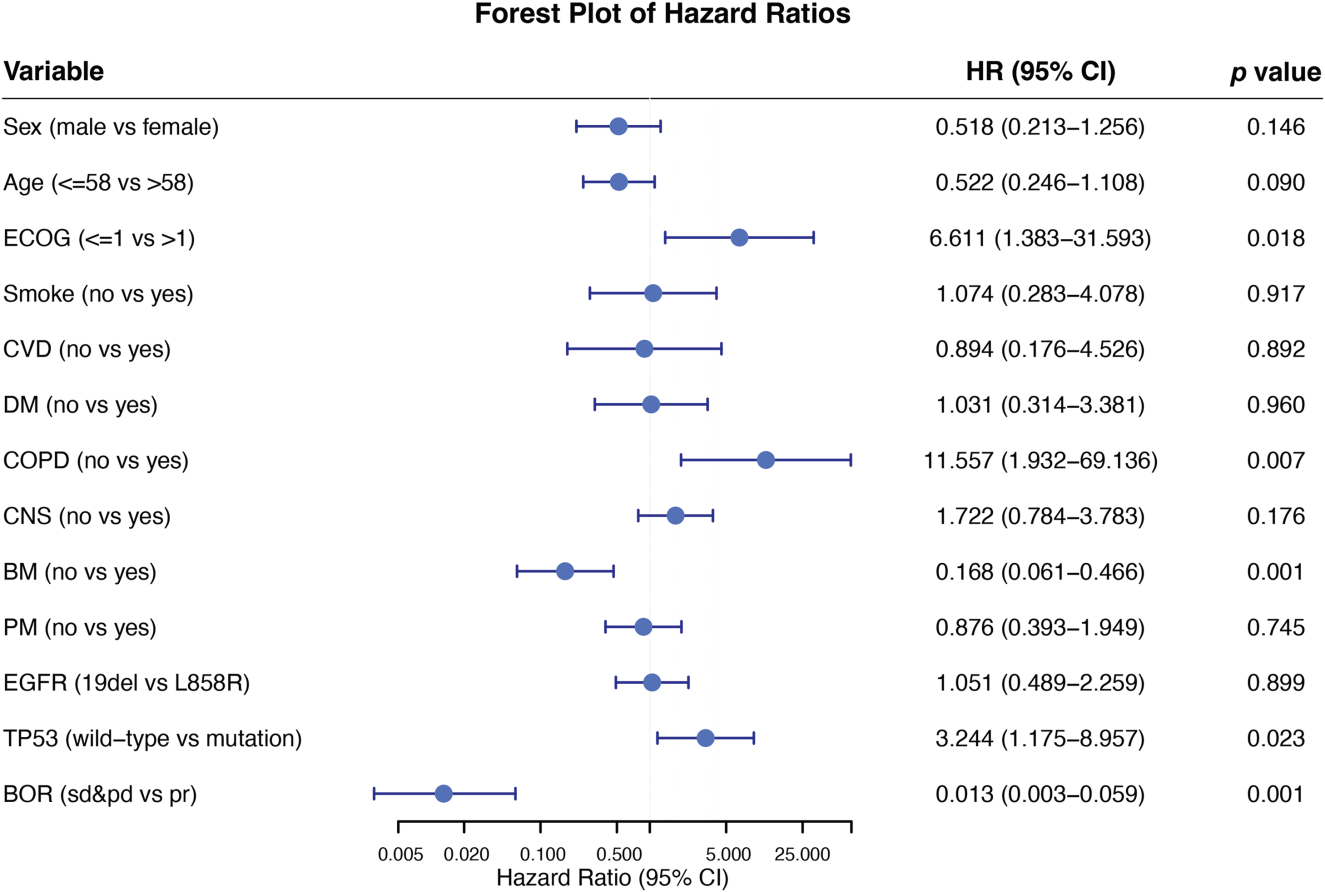
Subgroup analysis forest plot for progression-free survival

In this empirical study, the researcher assessed the best object response (BOR) of aumolertinib using patient historical records. The BOR indicated that 36 patients revealed a partial response, 9 demonstrated stable conditions, and 2 experienced progressive disease. The objective response rate (ORR) was 76.6% (36/47), while the disease control rate (DCR) was 95.7% (45/47). The ORR of patients with underlying brain metastases was 72.7% (16/22), while the DCR was 95.5% (21/22). At the study cutoff, 40 patients either suffered from disease progression or died due to their disease. Specifically, disease progression or mortality was observed in 77.8% (20/27) of patients with the 19del mutation and 94.1% (16/17) of those with the *L858R* mutation. The PFS analysis by additional subgroups including sex, age, smoking history, and baseline ECOG performance status, is presented in Fig. 6.

### 3.3 Safety of Treatment

At the data cutoff point, 66.0% (31/47) of cases (median follow-up, 48.1 months) were free from any treatment-related AEs. The retrospective analysis identified several AEs, which included rash (17.0%), skin decrustation (4.2%), pruritus (4.2%), dental ulcer (4.2%), increased creatine kinase (2.1%), and musculoskeletal aches (2.1%) as detailed in Table 2. Among the treatment-emergent AEs, decrustation of the skin (2.1%) and dental lesions (2.1%) were classified as Grades 3–4. A total of 33.3% (9/27) patients with 19del had AEs, and 35.3% (6/17) patients with *L858R* mutation had AEs. The prevalence of AEs was comparable between the two gene mutation types. The discontinuation and reduction of dosage due to drug toxicity were not observed. All adverse reactions were effectively alleviated through targeted symptomatic management, and no long-term toxicities related to the heart, lungs, etc. were observed in the real-world retrospective study.

**Table 2:** Adverse events (AEs)

Adverse Reactions	All Grades, No. **(%)**	Grade 1, No. **(%)**	Grade 2, No. **(%)**	Grades 3–4, No. **(%)**
Rash	8 (17.0)	5 (10.6)	3 (6.4)	0
Decrustation of skin	2 (4.2)	1 (2.1)	0	1 (2.1)
Pruritus	2 (4.2)	0	2 (4.2)	0
Dental ulcer	2 (4.2)	0	1 (2.1)	1 (2.1)
Increase creatine kinase	1 (2.1)	1 (2.1)	0	0
Musculoskeletal aches	1 (2.1)	0	1 (2.1)	0

CTCAE v5.0: Grade 1: Mild; either no symptoms or mild symptoms, with observation rather than intervention indicated. Grade 2: Moderate to severe effects, potentially requiring minimal, local or non-invasive treatment; potentially interfering with age-appropriate instrumental activities of daily living. Grade 3: Severe or medically significant effects that while not immediately life-threatening may need hospitalization or prolongation of existing hospitalization potentially disabling or leading to substantial impairment of self-care and daily living activities. Grade 4: Life-threatening requiring urgent intervention indicated.

## 4 Discussion

This study marks a pioneering multicenter clinical investigation in China, with a follow-up period exceeding four years for patients with *EGFR*-mutant NSCLC treated with aumolertinib. The findings indicate a favorable profile regarding efficacy and safety within the 1st-line treatment defense.

In the current study, a mPFS duration of 22.2 months was observed, which was higher than that of other published pivotal clinical trials [11,15,16]. Moreover, this study followed the trial’s secondary endpoints by demonstrating a 39.7-month mOS. This method temporarily solved the data gaps in the survival period [11]. The mPFS of this study was higher than that of commonly used third-generation tyrosine kinase inhibitors of the same class, which were 18.9 and 20.8 months, respectively, for osimertinib and furmonertinib [15,16]. In contrast to the AENEAS study, this study included a limited group of patients with rare mutations with improved survival times. Compared with the three Phase III clinical studies [11,15,16], this retrospective study reported a higher proportion of brain metastases (46.8%) at first diagnosis, indicating a poor prognosis. However, the current findings suggest that the mPFS for patients with brain metastasis was 19.7 months, exceeding the results observed in the AENEAS study. Despite the absence of statistically substantial variations in mPFS between both groups (*p* = 0.724), the subgroups characterized by brain metastasis displayed a trend towards shorter mPFS than those without brain metastasis (19.7 vs. 24.6 m). Pharmacokinetic studies in mice revealed that aumolertinib can pass the blood-brain barrier. Similarly, the present findings were aligned with previous studies on CNS metastases in *EGFR*-mutated advanced NSCLC [17]. The Grades 3–4 treatment-associated AEs were decrustation of the skin (2.1%) and dental ulcers (2.1%). No AE-related dose modifications were observed.

In previous studies [18–20], patients with *L858R* had a worse prognosis than those with exon 19del. This study examined the variations in mPFS across various mutation subtypes. The current results depicted that *L858R* mutated patients demonstrated a substantially reduced mPFS compared to those with the 19del mutation (15.2 vs. 28.4 m), with a statistically significant disparity observed between both subgroups (*p* = 0.048). Both subgroups revealed statistical variances consistent with previous studies [18,19]. Currently, several combination regimens comprising TKIs have shown improved outcomes for the *L858R* subtype in phase III clinical studies [21,22], and TKI remains the standard 1st-line treatment option due to safety concerns. However, therapeutic options following disease progression after first-line treatment remain relatively limited. Amivantamab, a bispecific antibody targeting both EGFR and MET, has demonstrated promising efficacy in cases with disease progression following osimertinib treatment [23]. Simultaneously, Amivantamab has been found effective in patients harboring EGFR exon 20 insertion mutations [24].

Osimertinib with chemotherapy as 1st-line treatment has shown initial efficacy in patients with *L858R* or CNS metastasis [25]. Strategies for combination therapy are still being explored. The presence of concomitant mutations is a definitive factor affecting the effectiveness of TKI in aNSCLC characterized by *EGFR* mutations [26]. *TP53* alterations have found to be associated with more rapid drug resistance, irrespective of the underlying mechanisms, resulting in decreased PFS and OS in patients with *EGFR*-mutant NSCLC [27]. Mutant *TP53* has greater stability than WT *TP53*, leading to its functional loss. This accumulation within cells promotes cancer cell growth, penetration, metastasis, and resistance to therapeutic agents [28]. *TP53* was one of the most prevalent concomitant mutations in the NGS data of this research, although the NGS panels of patients were not fully consistent in this real-world retrospective study (32/47, 68.1%). The mPFS trend of patients with the *TP53* mutation was shorter than that of *TP53* WT patients (19.2 vs. 31.6 m), but no considerable disparity was observed between both groups (*p* = 0.175). Subgroup analysis revealed that a higher Eastern Cooperative Oncology Group Performance Status (ECOG) score, the presence of chronic obstructive pulmonary disease (COPD), and TP53 mutation status were markedly linked with reduced PFS. Conversely, achieving the best objective response was strongly linked to prolonged PFS.

In this study, a patient with stage IV SCC was found to have a 19del and multiple co-mutations, including *TP53* via NGS. The patient’s ECOG performance status was 4 at diagnosis. The patient’s symptoms resolved rapidly after administering aumolertinib, and computed tomography (CT) confirmed a partial remission, with a PFS of 19.8 months. Despite rare *EGFR* mutations, like *EGFR KDD* and *EGFR S768I*, two patients had favorable PFS of 24.6 and 28.4 m, respectively. The patient with the *EGFR KDD* mutation also harbored the *TP53* concomitant mutations. Many studies have demonstrated that the therapeutic efficacy of 1st and 2nd generation TKIs is restricted in patients with lung SCC and lung adenocarcinoma that contains *EGFR KDD* mutations [29–31]. Until now, there has been relatively limited data concerning treating such patients with 3rd generation TKIs and further research is warranted.

The most common AEs in the current cohort were rash (17.0%), skin decrustation (4.2%), pruritus (4.2%), dental ulcer (4.2%), increased creatine kinase (2.1%), and musculoskeletal aches (2.1%). The prevalence of grade 3 or higher AEs was relatively low (4.2%), and no treatment-associated interstitial lung disease was depicted. In the AENEAS study, which followed multiple system-related AEs, 2 patients (0.9%) had treatment-related interstitial lung disorder [11]. The most common AEs of osimertinib are leukopenia, anemia, thrombocytopenia, diarrhea, neutropenia, and weight loss [15]. The most common adverse reactions of furmonertinib include elevated alanine aminotransferase, diarrhea, elevated aspartate aminotransferase, rash, etc. [16]. However, no new AEs related to the hematological system or diarrhea were observed in this study and no long-term toxicity was observed in relation to the heart, lungs, etc. It was also predicted that the study failed to show significant differences consistent with previous studies due to the small (limited) sample size.

This study has several limitations. The retrospective nature of the study and small (limited) sample size may have influenced the interpretation of the efficacy and safety results. The current toxicity evaluation was partly based on telephone follow-ups, potentially affecting the accuracy of recording adverse reactions relative to the prospective clinical trials.

## 5 Conclusion

This study retrospectively analyzed the long-term efficacy and safety of first-line aumolertinib for treating advanced NSCLC with EGFR mutations in the real world setting. Our research revealed that patients with the L858R mutation demonstrated poorer prognosis compared to those with the exon 19 deletion. Despite the small sample size, we still observed a trend toward poor prognosis in patients with brain metastases and TP53 mutations. First-line treatment with aumolertinib for EGFR-mutated NSCLC demonstrated safety and efficacy, which were consistent with other EGFR TKIs. Additionally, it had a relatively low incidence of adverse events in the real world.

## Data Availability

The original data underpinning the conclusion of this article will be provided by the corresponding author as needed.
